# Sensitivity to Thyroid Hormones and Risk of Prediabetes: A Cross-Sectional Study

**DOI:** 10.3389/fendo.2021.657114

**Published:** 2021-05-04

**Authors:** Bingyang Liu, Zixiao Wang, Jinrong Fu, Haixia Guan, Zhaohui Lyu, Wei Wang

**Affiliations:** ^1^ Department of Endocrinology, Shengjing Hospital of China Medical University, Shenyang, China; ^2^ Department of Physical Examination Center, The First Hospital of China Medical University, Shenyang, China; ^3^ Department of Endocrinology and Metabolism, The First Hospital of China Medical University, Shenyang, China; ^4^ Department of Endocrinology, Guangdong Provincial People’s Hospital, Guangdong Academy of Medical Sciences, Guangzhou, China; ^5^ Department of Endocrinology, The First Medical Center of Chinese PLA General Hospital, Beijing, China

**Keywords:** prediabetes, hyperglycemia, thyroid hormone sensitivity, resistance to thyroid hormone, metabolism

## Abstract

**Context:**

Thyroid hormone influences glucose homeostasis through central and peripheral regulations. To date, the link between sensitivity to thyroid hormones and prediabetes remains unknown. We aimed to investigate the association between thyroid hormones sensitivity and risk of prediabetes in both general and euthyroid populations.

**Methods:**

Participants with serum free triiodothyronine (FT3), free thyroxine (FT4), and thyrotropin (TSH) measurements from the health checkup programs of the First Hospital of China Medical University were collected. We measured the parameters representing central and peripheral sensitivities to thyroid hormones (central sensitivity, assessed by calculating Thyroid Feedback Quantile-based Index (TFQI), TSH Index (TSHI), and Thyrotroph Thyroxine Resistance Index (TT4RI); peripheral sensitivity, evaluated by FT3/FT4 ratio). Associations between thyroid hormones sensitivities and risk of prediabetes were assessed with logistic regression.

**Results:**

A total of 4378 participants (mean age ± SD, 49 ± 11 years) were included, with 1457 (33%) subjects had prediabetes. The risk of prediabetes was negatively associated with levels of TSHI (odds ratio [OR] 0.91; 95% confidence interval [CI], 0.85–0.97), TT4RI (OR 0.91; 95% CI, 0.84–0.99) and Parametric TFQI (PTFQI) (OR 0.89; 95% CI, 0.83–0.95) among all subjects. The association remained significant in euthyroid subjects and euthyroid subjects with negative thyroid autoimmunity. Higher FT3/FT4 ratio was associated with a mild increased risk of prediabetes (95% CI 1.09; 1.02–1.16). Compared with subjects in the lowest quartile of PTFQI, those in the highest quartile had lower risk of prediabetes (0.70; 95% CI, 0.58–0.84).

**Conclusions:**

Decreased central sensitivity to thyroid hormones is associated with lower risk of prediabetes. This demonstrates the complex interaction between thyroid system and glucose metabolism. Future studies are warranted to confirm our findings and underlying mechanisms.

## Introduction

Prediabetes is a condition of intermediate hyperglycemia below the diagnostic threshold of type 2 diabetes mellitus (T2DM). Higher risk for nephropathy and cardiovascular disease has been found in patients with prediabetes, suggesting the pathogenic effects of impaired glucose may start at this early stage of diabetes (1).With aging, urbanization and changes in lifestyles, the population of prediabetes has been rising worldwide. The prevalence of prediabetes in China has increased from 15.5% in 2008 (2) to 35.2% in 2017 (3), which has become a major burden for public health.

Nearly 70% of patients with prediabetes will develop diabetes eventually (4). To prevent and treat diabetes in early stage, many studies aimed to find risk factors for prediabetes and diabetes. Thyroid hormone is an important regulator of glucose and lipid metabolism. It is well established that patients with thyroid disorders were more likely to develop obesity, metabolic syndrome and diabetes than healthy individuals (5). Both hypothyroidism and hyperthyroidism has been suggested to be associated with diabetes (6–8). Furthermore, even variations of thyroid function within normal range might be associated with development and progression of diabetes (9). These contradictory findings from previous studies suggest there are more complex pathophysiologic mechanisms linking the relationship between thyroid hormones and glucose metabolism.

The homeostasis of thyroid system can be achieved through the coordination of negative feedback regulation in the central nervous system, and a set of iodothyronine deiodinase (DIO) and thyroid hormone receptor (THR) in peripheral target organs (5). Recently, a study suggests that reversible central thyroid hormone resistance, which existed both in thyroid hormone overproduction and deficiency, was related to risks of diabetes and diabetes-related mortality (10). It is uncertain whether changes of sensitivity to thyroid hormones impact the development of prediabetes. Therefore, in this population-based study, we aimed to assess the associations of sensitivity to thyroid hormones with the risk of prediabetes in the general and euthyroid subjects.

## Methods

### Study Population

7,688 participants with thyroid function measurements in health checkup programs of the First Hospital of China Medical University from January 2017 to December 2018 were included consecutively. Among 4,976 individuals with complete baseline information, subjects with incorrect data (n = 5) and known diabetes (n = 593) were excluded.

### Assessment of Thyroid Function

Serum free triiodothyronine (FT3), free thyroxine (FT4), thyrotropin (TSH), and thyroid peroxidase antibodies (TPOAb) were measured with the automated immunochemiluminescent assay (ICMA) kits (Abbott, IL, USA). The reference ranges of TSH, FT4 and FT3 were 0.35 to 4.94 mIU/L, 9.01 to 19.05 pmol/L, and 2.63 to 5.70 pmol/L, respectively. Euthyroid was defined as having FT3, FT4 and TSH within normal ranges, and without known thyroid disease history. Thyroid autoimmunity was defined as TPOAb ≥ 5.61 IU/ml.

### Baseline and Other Measurements

Body weight, height, waist circumference (WC), and blood pressure were measured according to standard protocols. Body mass index (BMI) was calculated by dividing body weight in kg by the square of height in meters. Hypertension was defined by self-report of hypertension or elevated blood pressure (systolic blood pressure ≥ 140 mm Hg or diastolic blood pressure ≥ 90 mm Hg).

Venous blood samples were collected in the morning between 7:00 am and 10:00 am after an 8- to 12-h fast, and then promptly centrifuged and analyzed. The concentrations of total cholesterol (TC), triglyceride (TG), low-density lipoprotein cholesterol (LDL-C), high-density lipoprotein cholesterol (HDL-C), fasting plasma glucose (FPG), and glycosylated hemoglobin (HbA_1c_) were determined by enzymatic methods on the Automatic Biochemical Analyzer. Dyslipidemia was defined as TG ≥ 1.7 mmol/L or TC ≥ 5.2 mmol/L or LDL ≥ 3.4 mmol/L or HDL ≤ 1.0 mmol/L. Prediabetes was defined as impaired fasting glucose (IFG, FPG 5.6 mmol/L to 6.9 mmol/L) or HbA_1c_ 5.7% to 6.4% (11).

### Indices of Thyroid Hormone Sensitivity

Central indices of thyroid hormone sensitivity included TSH index (TSHI), TSH T4 resistance index (TT4RI), Thyroid Feedback Quantile-based Index (TFQI), and Parametric Thyroid Feedback Quantile-based Index (PTFQI). TSH index, TSHI = ln TSH (mIU/L) + 0.1345 * FT4 (pmol/L) (12). TSH T4 resistance index, TT4RI = FT4 (pmol/L) * TSH (mIU/L) (13). TFQI and PTFQI were calculated using the algorithm developed by Laclaustra et al. (10). FT3/FT4 ratio was calculated to evaluate the peripheral thyroid hormone sensitivity.

### Statistical Analysis

Continuous variables with normal distribution were expressed as mean ± SD, and those with skewed distribution were described as median (interquartile ranges, IQRs). Categorical variables were expressed as number of events and percentages. Comparisons between characteristics of participants with and without prediabetes were conducted using the independent samples t-test or Mann-Whitney U test.

We investigated the odds ratio (OR) of prediabetes with per SD change of indices of thyroid hormone sensitivity in three adjusted models. Model 1 was adjusted for age and gender; model 2 was adjusted for age, gender and BMI; model 3 was adjusted for age, gender, BMI, WC, hypertension, and dyslipidemia. Variables in the multivariable models represent the most common confounding or mediating factors of the association between resistance to thyroid hormone and prediabetes. Additionally, we performed the following sensitivity analysis: restricting analyses to euthyroid participants; excluding participants with thyroid dysfunction and thyroid autoimmunity. Stratification by gender, age categories (cutoff age 60 years) and BMI groups (divided by BMI < 24.0, 24.0 ≤ BMI < 28 or BMI ≥ 28.0) was performed.

Next, we compared the risk of IFG, HbA_1c_ 5.7% to 6.4% and overall prediabetes across quartiles of indices of thyroid hormone sensitivity using the logistic regression analysis, with the first quartile as the reference group. The results of logistic regression analysis were expressed as odds ratio (OR) and 95% confidence interval (CI). *P*-values < 0.05 were considered significant. Statistical analyses were conducted using IMB SPSS, version 26 and STATA version 16 for Windows.

## Results

### Baseline Characteristics

Baseline characteristics of 4,378 eligible participants are presented in **Table 1**. The prevalence of prediabetes was 33%. The mean age of included participants was 49 years and 53% were men. Compared to participants with normoglycemia, participants with prediabetes were more likely to be older, men, with higher prevalence of hypertension and dyslipidemia. Participants with prediabetes tended to have higher BMI, WC, and FT3/FT4, while the levels of TSHI, TT4RI, and PTFQI were significantly lower.

**Table 1:** Baseline characteristics of participants with normoglycemia and prediabetes.

	Overall (4,378)	Normoglycemia (2,921, 67%)	Prediabetes (1,457, 33%)	P
Age (y)	49 ± 11	47 ± 10	53 ± 10	<0.001
Men, %	2,317, 53%	1,501, 51%	816, 56%	0.004
BMI (kg/m^2)^	25.2 3.4	24.8 ± 3.4	26.0 ± 3.2	<0.001
WC (cm)	84 (77, 91)	83 (76, 90)	87 (80, 94)	<0.001
Hypertension, %	1493, 34%	845, 29%	648, 45%	<0.001
Dyslipidemia, %	2771, 63%	1700, 58%	1071, 74%	<0.001
FPG (mmol/L)	5.1 ± 0.5	4.9 ± 0.4	5.5 ± 0.5	<0.001
HbA_1c_ (%)	5.4 ± 0.4	5.2 ± 0.3	5.8 ± 0.3	<0.001
FT3 (pmol/L)	4.36 (4.04, 4.69)	4.36 (4.04, 4.69)	4.37 (4.05, 4.69)	0.554
FT4(pmol/L)	13.33 ± 1.85	13.35 ± 1.83	13.28 ± 1.89	0.233
TSH (mIU/L)	1.64 (1.16, 2.37)	1.68 (1.19, 2.42)	1.61 (1.12, 2.28)	0.001
FT3/FT4	0.33 ± 0.05	0.33 ± 0.05	0.34 ± 0.05	0.021
TSHI	2.25 ± 0.76	2.28 ± 0.76	2.20 ± 0.77	0.001
TT4RI	21.76 (15.29, 31.52)	22.32 (15.72, 32.01)	20.80 (14.32, 30.50)	<0.001
TFQI	0.03 (−0.54, 0.59)	0.06 (−0.52, 0.60)	−0.02 (−0.58, 0.59)	0.074
PTFQI	0.004 ± 0.302	0.017 ± 0.300	−0.021 ± 0.305	<0.001

BMI, body mass index; WC, waist circumference; FPG, fasting plasma glucose; HbA1c, glycated hemoglobin; FT3, free triiodothyronine; FT4, free thyroxine; TSH, thyroid stimulating hormone; TSHI, TSH index; TT4RI, TSH T4 resistance index; TFQI, Thyroid Feedback Quantile-based Index; PTFQI, Parametric Thyroid Feedback Quantile-based Index.

### Association of Sensitivity to Thyroid Hormones With the Risk of Prediabetes

The adjusted association between prediabetes with resistance to thyroid hormone indices was shown in **Table 2**. Higher level of TSHI, TT4RI, and PTFQI were associated with lower levels of prediabetes. With per SD increase of TSHI, TT4RI, and PTFQI, the odds ratio of prediabetes was 0.91 (95% CI, 0.85–0.97), 0.91 (95% CI, 0.84–0.99), and 0.89 (95% CI, 0.83–0.95), respectively. The association remained significant after adjustments for age, gender, BMI, WC, hypertension, and dyslipidemia. There was no significant association between the level of TFQI and prediabetes. Increased FT3/FT4 ratio was associated with higher risk of prediabetes (OR, 1.09; 95% CI, 1.02–1.16), whereas the association was insignificant in the fully-adjusted model.

**Table 2:** Association between indices of thyroid hormone sensitivity and prevalence of prediabetes.

OR (95% CI)					
	TSHI (+1 SD)	TT4RI (+1 SD)	TFQI (+1 SD)	PTFQI (+1 SD)	FT3/FT4 (+1 SD)
General (n = 4,378, 33%)					
Model 1	0.91 (0.85–0.97) ^**^	0.91 (0.84–0.99) ^*^	0.94 (0.88–1.01)	0.89 (0.83–0.95) ^***^	1.09 (1.02–1.16) ^*^
Model 2	0.90 (0.84–0.96) ^**^	0.89(0.81–0.97) ^*^	0.96 (0.90–1.03)	0.89 (0.83–0.95) ^**^	1.06 (0.99–1.13)
Model 3	0.88 (0.83–0.94) ^***^	0.88 (0.80–0.97) ^**^	0.96 (0.90–1.02)	0.88 (0.82–0.94) ^***^	1.05 (0.98–1.12)
Euthyroid (n = 4073, 33%)					
Model 1	0.83(0.75–0.92) ^**^	0.78 (0.68–0.91) ^**^	0.93 (0.87–1.00) ^*^	0.88 (0.82–0.95) ^***^	1.09 (1.02–1.18) ^*^
Model 2	0.81 (0.73–0.90) ^***^	0.76 (0.65–0.88) ^***^	0.95 (0.88–1.02)	0.88 (0.82–0.95) ^**^	1.06 (0.98–1.14)
Model 3	0.80 (0.72–0.89) ^***^	0.74 (0.64–0.86) ^***^	0.94 (0.88–1.01)	0.87 (0.81–0.94) ^***^	1.05 (0.98–1.14)
Euthyroid participants with negative TPOAb (n = 3,481, 33%)					
Model 1	0.85 (0.75–0.95) ^**^	0.79 (0.67–0.94) ^**^	0.91 (0.84–0.98) ^*^	0.88 (0.82–0.95) ^**^	1.12 (1.03–1.21) ^**^
Model 2	0.82 (0.73–0.92) ^**^	0.76 (0.64–0.90) ^**^	0.92 (0.86–1.00) ^*^	0.88 (0.82–0.95) ^**^	1.08(0.99–1.18)
Model 3	0.81 (0.72–0.91) ^***^	0.74 (0.62–0.88) ^**^	0.92 (0.85–0.99) ^*^	0.87 (0.81–0.94) ^***^	1.08 (0.99–1.18)

Logistic regression models: model 1, adjusted for age and gender; model 2, adjusted for age, gender and BMI; model 3, adjusted for age, gender, BMI, WC, hypertension and dyslipidemia.*P <0.05; **P <0.01; ***P <0.001.

The association between TSHI, TT4RI and prediabetes was more significant among euthyroid participants and negative thyroid autoimmunity. Besides, TFQI was negatively associated with the risk of prediabetes in euthyroid participants with negative thyroid autoimmunity (OR, 0.92; 95% CI, 0.85–0.99) in the fully adjusted model. Stratification analyses for gender, age, and BMI categories did not show significantly differential risks of prediabetes (*P*_for interaction_ for all analyses > 0.05) (**Supplementary Table 1**).

### Association of Prediabetes With Sensitivity to Thyroid Hormones in Quartiles

**Figure 1:**
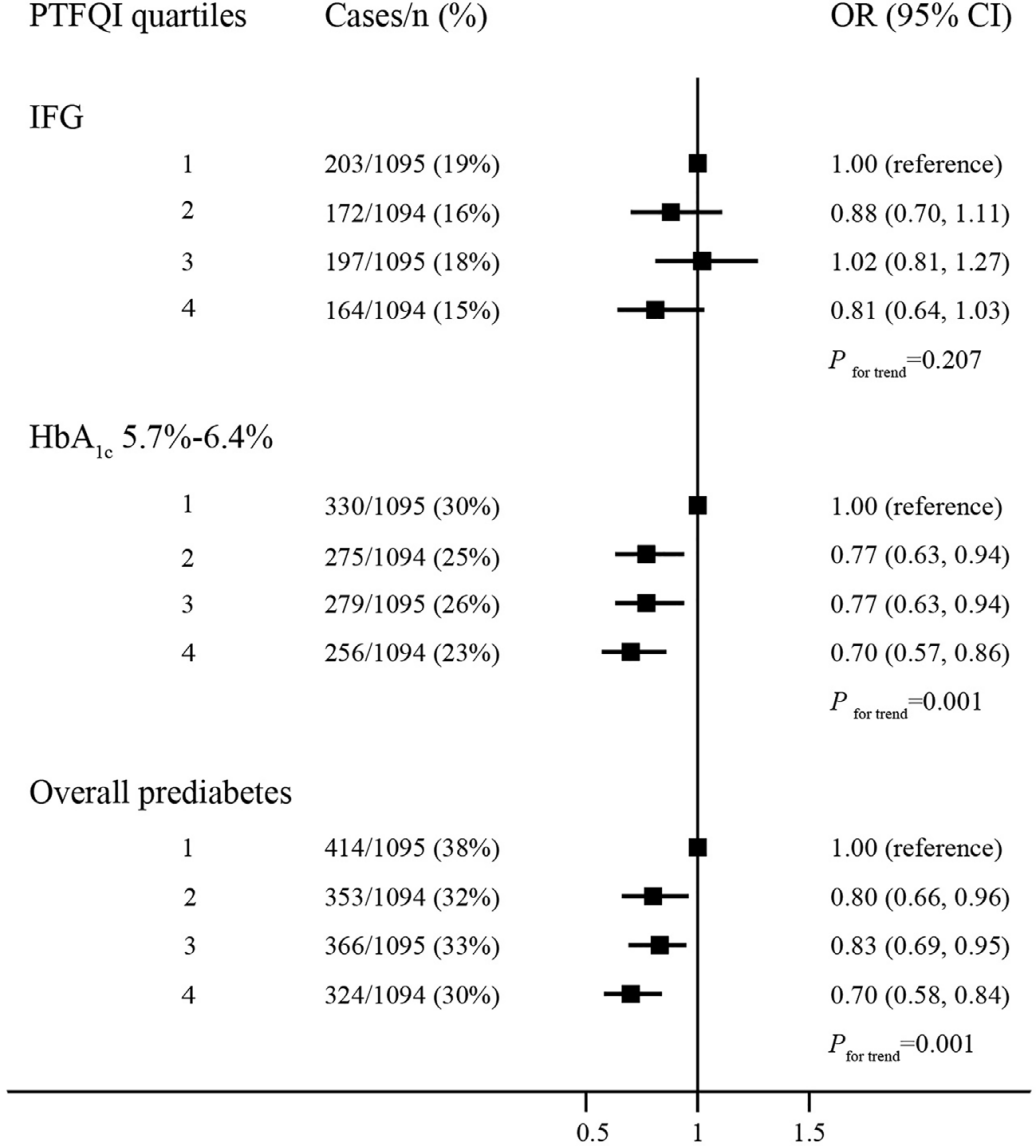
The association between quartiles of PTFQI and risk or prediabetes. Logistic regression model; models are adjusted for age, gender, BMI, WC, hypertension and dyslipidemia.

The prevalence of elevated HbA_1c_ and overall prediabetes decreased significantly across quartiles of PTFQI (**Figure 1**). Compared with the lowest quartile of PTFQI, the risks of elevated HbA_1c_ and overall prediabetes in participants with highest quartile of PTFQI were 0.70 (95% CI, 0.57–0.86) and 0.70 (95% CI, 0.58–0.84), respectively. However, there was no significant association between IFG and quartiles of PTFQI. Increased quartiles of TSHI, TT4RI, and TFQI were also associated with lower risk of IFG, elevated HbA_1c_, and overall prediabetes to different extent (**Supplementary Tables 2**–**4**). Neither individual events of prediabetes nor overall prediabetes were associated with quartiles of FT3/FT4 ratio (**Supplementary Figure 1**).

## Discussion

In this population-based cross-sectional study, we found decreased central thyroid hormone sensitivity (increased TSHI, TT4RI, and PTFQI) were associated with lower risk of prediabetes. The relationship remained consistent after excluding participants with thyroid dysfunction and thyroid autoimmunity. In addition, increased peripheral thyroid hormone sensitivity (increased FT3/FT4 ratio) was correlated to higher risk of prediabetes, while the correlation was insignificant after adjusting for multiple confounding factors. To our knowledge, this is the first study evaluating the association between sensitivity to thyroid hormones and prediabetes.

Thyroid hormones have profound effects on the regulation of glucose homeostasis (5). Most of previous studies found increased prevalence of T2DM and prediabetes among subjects with hypothyroidism (6, 9, 14–16). A 7-year prospective study of Korean population reported that the risk of T2DM was positively related with the level of TSH, while negatively associated with the level of T3 and FT4 (14). Decreased level of FT4 within normal range was one of risk factors for hyperglycemia and insulin resistance (16). However, a study of Germany and Danish population suggested that the level of thyroid hormones were positively associated with prevalent and incident T2DM (7). Two cross-sectional studies reported a positive correlation between the level of FT4 and FPG (17, 18). Inconsistency in previous studies highlights that TSH or thyroid hormone levels alone maybe insufficient to explain the relationship between thyroid system and glycemic dysregulation.

Circulating thyroid hormones are regulated by the hypothalamus–pituitary–thyroid (HPT) axis through a negative feedback mechanism. Dysregulation of HPT axis often coexisted with metabolic syndrome or diabetes, a condition well-known in the elderly population (19). In 2009, Jostel et al. first proposed the calculation of TSHI for estimating the sensitivity of pituitary to thyroid hormones (20). Impaired HPT sensitivity to thyroid hormones has been suggested to be related to multiple unfavorable clinical outcomes, including obesity (21), T2DM (10, 22), and decreased estimated glomerular filtration rate (eGFR) (22). In 2019, Laclaustra et al. reported the association between TFQI and the risk of diabetes, obesity and diabetes-related deaths prospectively (10). These finding provides explanations for the association of both hyperthyroidism and hypothyroidism with metabolic disease. Consistently, a recent cross-sectional study suggests that decreased sensitivity to thyroid hormones were positively related to the level of adipocyte fatty acid-binding protein (A-FABP) (23). However, to our knowledge, there has been no study focusing on the relationship between sensitivity to thyroid hormones and the risk of prediabetes. Contrary to most previous studies, our result showed decreased sensitivity to thyroid hormones has a protective effect on prediabetes, which was consistent in subgroup analyses among different age, gender, and BMI categories. A cross-sectional study in China also found a negative association between HbA_1c_ and TSHI, though it did not reach statistical significance (24). The contradictory results from the aforementioned studies could be attributed to differences in age, gender, ethnicity, and iodine status of study population. Taken all into consideration, these evidences suggest the potential role of sensitivity to thyroid hormones in the development of metabolic disease.

There are several pathways that may explain the observed association between indices of central resistance to thyroid hormones and prediabetes. Firstly, thyroid hormone plays an important role in insulin signaling pathway (25).The level of FT3 and FT4 has been reported to be negatively associated with insulin resistance index (HOMA-IR), while TSH was positively associated with HOMA-IR (26, 27). In patients with hypothyroidism, impaired translocation of GLUT4 glucose transporters contributed to decreased insulin-stimulated rates of glucose transport (28). Besides, a decreased level of FT4 within the normal range was correlated with risk of insulin resistance (29, 30). Participants with decreased central sensitivity of thyroid hormones tended to have increased FT4 levels, which may decrease risk of prediabetes by improving insulin sensitivity and glucose utilization. Secondly, thyroid hormone can regulate glucose homeostasis at genetic levels by affecting transcription factors involved in adipogenesis (31), genes regulating insulin resistance (32), and beta-cell proliferation (33). Mice with resistance to thyroid hormone (RTH) exhibited impaired THR signaling, leading to reduced hepatic glucose production and reduced gluconeogenesis (34). However, this has lead to an inconsistency, as our study found that glucose tolerance is actually higher in subjects with increased central thyroid hormone resistance. Given the cross-sectional design of our study, it is possible that glucose metabolism can lead to the reversed changes in thyroid physiology, but more direct evidence is warranted in future studies. Thirdly, the cross-talk between HPT axis and glucose metabolism may also be achieved through hormonal regulation. Leptin is a hormone predominantly secreted from adipose tissue, which acts on hypothalamic appetite center to regulate the caloric intake and energy storage (35). The level of leptin has been reported to change in status of thyroid dysfunction both in animal models and human beings (36). Central thyroid hormone sensitivity may change the secretion of leptin, affecting the feeding behavior, leading to the change of adiposity, and glucose metabolism. However, the exact modulating mechanisms between central thyroid hormone sensitivity and leptin pathway are unclear.

Besides the HPT axis, the relation between thyroid function and glucose metabolism could also be modulated by peripheral DIO activity. Inactive T4 is transformed into active T3 by DIO1 in the peripheral tissues (36). In adipose tissue of obese subjects, the expression of DIO1 was upregulated through the stimulation of leptin (37). Several previous studies reported that increased FT3 and FT3/FT4 ratio were associated with unfavorable metabolic phenotypes, including higher BMI, TC, BP, and FPG (38–40). Our study found a higher conversion of FT4 to FT3 in patients with prediabetes, though it did not reach significance after adjusting for BMI, WC, dyslipidemia, and hypertension. And a large cross-sectional study in China suggested that the level of FT3 and FT3/FT4 ratio were negatively associated with the risk of T2DM, independent of multiple confounding factors (17). This discrepancy suggests the role of thyroid hormones in metabolic disorders changes with different types and stages of disease. Only a few cross-sectional studies existed investigating the association between FT3/FT4 ratio and risk of metabolic disease, and the mechanisms of regulation remained unknown.

The strengths of this study include the large number of participants and the detailed information of available covariates for adjustment analysis. We also performed secondary analyses among euthyroid participants and individuals with negative thyroid autoimmunity. However, our study has several limitations. Firstly, the cross-sectional design based on a single measurement is insufficient to establish a cause and effect relationship between sensitivity to thyroid hormones and prediabetes. Though we have adjusted for multiple confounding factors, the possibility of reverse causation and influence of medication cannot be ruled out. Secondly, the prevalence of dyslipidemia in this cohort was higher than previous studies, and may therefore not be generalizable to other populations. Thirdly, medication history of our subjects was absent. Metformin could increase the inhibitory effect of thyroid hormones on central TSH secretion by modulating the expression of THRs or DIO2 at hypothalamic-pituitary level, leading to decreased levels of TSH without significant changes of FT4 (12). It is likely that participants with prediabetes in our study have started on metformin regimens, leading to increased pituitary sensitivity in this group. However, the majority of our subjects were euthyroid, whose TSH levels were less likely to be influenced by this antidiabetic agent (12, 41). It is important to emphasize that thyroid resistance indices cannot replace the evaluation of thyroid function and THR mutation test, but rather a supplementary method to learn the association between thyroid system and other metabolic disease.

In conclusion, our study demonstrates the association between increased central resistance to thyroid hormones and lower risk of prediabetes. This study provides evidence for the significance of thyroid hormones in their interactions with glucose metabolism, while future studies are warranted to confirm these findings and underlying mechanism.

## Data Availability Statement

The original contributions presented in the study are included in the article/**Supplementary Material**. Further inquiries can be directed to the corresponding authors.

## Ethics Statement

The studies involving human participants were reviewed and approved by The Ethics Committee of China Medical University. The patients/participants provided their written informed consent to participate in this study.

## Author Contributions

BL carried out the study, collected and analyzed data, and wrote the paper. ZW facilitated collecting subjects’ information. JF and HG helped analyzed and draft the manuscript. As the corresponding authors, ZL and WW contributed equally to conceive the study design, supervise the study, and revise the manuscript. All authors contributed to the article and approved the submitted version.

## Conflict of Interest

The authors declare that the research was conducted in the absence of any commercial or financial relationships that could be construed as a potential conflict of interest.

## References

[1] Ali MK Bullard KM Saydah S Imperatore G Gregg EW. Cardiovascular and Renal Burdens of Prediabetes in the USA: Analysis of Data From Serial Cross-Sectional Surveys, 1988–2014. Lancet Diabetes Endocrinol (2018) 6:392–403. 10.1016/S2213-8587(18)30027-5 29500121 PMC6615033

[2] Yang W Lu J Weng J Jia W Ji L Xiao J. Prevalence of Diabetes Among Men and Women in China. N Engl J Med (2010) 362:1090–101. 10.1056/NEJMoa0908292 20335585

[3] Li Y Teng D Shi X Qin G Qin Y Quan H. Prevalence of Diabetes Recorded in Mainland China Using 2018 Diagnostic Criteria From the American Diabetes Association: National Cross Sectional Study. BMJ (2020) 369:m997. 10.1136/bmj.m997 32345662 PMC7186854

[4] Tabák AG Herder C Rathmann W Brunner EJ Kivimäki M. Prediabetes: A High-Risk State for Diabetes Development. Lancet (2012) 379:2279–90. 10.1016/S0140-6736(12)60283-9 PMC3891203 22683128

[5] Biondi B Kahaly GJ Robertson RP. Thyroid Dysfunction and Diabetes Mellitus: Two Closely Associated Disorders. Endocr Rev (2019) 40:789–824. 10.1210/er.2018-00163 30649221 PMC6507635

[6] Gronich N Deftereos SN Lavi I Persidis AS Abernethy DR Rennert G. Hypothyroidism is a Risk Factor for New-Onset Diabetes: A Cohort Study. Diabetes Care (2015) 38:1657–64. 10.2337/dc14-2515 26070591

[7] Ittermann T Schipf S Dorr M Thuesen BH Jorgensen T Volzke H. Hyperthyroxinemia is Positively Associated With Prevalent and Incident Type 2 Diabetes Mellitus in Two Population-Based Samples From Northeast Germany and Denmark. Nutr Metab Cardiovasc Dis (2018) 28:173–9. 10.1016/j.numecd.2017.10.016 29239740

[8] Jang J Kim Y Shin J Lee SA Choi Y Park EC. Association Between Thyroid Hormones and the Components of Metabolic Syndrome. BMC Endocr Disord (2018) 18:29. 10.1186/s12902-018-0256-0 29783969 PMC5963056

[9] Chaker L Ligthart S Korevaar TI Hofman A Franco OH Peeters RP. Thyroid Function and Risk of Type 2 Diabetes: A Population-Based Prospective Cohort Study. BMC Med (2016) 14:150. 10.1186/s12916-016-0693-4 27686165 PMC5043536

[10] Laclaustra M Moreno-Franco B Lou-Bonafonte JM Mateo-Gallego R Casasnovas JA Guallar-Castillon P. Impaired Sensitivity to Thyroid Hormones is Associated With Diabetes and Metabolic Syndrome. Diabetes Care (2019) 42:303–10. 10.2337/dc18-1410 30552134

[11] American Diabetes Association. 2. Classification and Diagnosis of Diabetes: Standards of Medical Care in Diabetes-2020. Diabetes Care (2020) 43:S14–31. 10.2337/dc20-S002 31862745

[12] Cappelli C Rotondi M Pirola I Agosti B Gandossi E Valentini U. TSH-Lowering Effect of Metformin in Type 2 Diabetic Patients: Differences Between Euthyroid, Untreated Hypothyroid, and Euthyroid on L-T4 Therapy Patients. Diabetes Care (2009) 32:1589–90. 10.2337/dc09-0273 PMC2732148 19502536

[13] Yagi H Pohlenz J Hayashi Y Sakurai A Refetoff S. Resistance to Thyroid Hormone Caused by Two Mutant Thyroid Hormone Receptors Beta, R243Q and R243W, With Marked Impairment of Function That Cannot be Explained by Altered In Vitro 3,5,3’ Triiodothyroinine Binding Affinity. J Clin Endocrinol Metab (1997) 82:1608–14. 10.1210/jcem.82.5.3945 9141558

[14] Jun JE Jee JH Bae JC Jin SM Hur KY Lee MK. Association Between Changes in Thyroid Hormones and Incident Type 2 Diabetes: A Seven-Year Longitudinal Study. Thyroid (2017) 27:29–38. 10.1089/thy.2016.0171 27809684

[15] Jun JE Jin SM Jee JH Bae JC Hur KY Lee MK. TSH Increment and the Risk of Incident Type 2 Diabetes Mellitus in Euthyroid Subjects. Endocrine (2017) 55:944–53. 10.1089/thy.2016.0171 28042645

[16] Gu L Yang J Gong Y Ma Y Yan S Huang Y. Lower Free Thyroid Hormone Levels are Associated With High Blood Glucose and Insulin Resistance and Normalize With the Metabolic Improvement in Type 2 Diabetic Mellitus With Euthyroid. J Diabetes (2020) 13:318–29. 10.1111/1753-0407.13118 32981234

[17] Gu Y Li H Bao X Zhang Q Liu L Meng G. The Relationship Between Thyroid Function and the Prevalence of Type 2 Diabetes Mellitus in Euthyroid Subjects. J Clin Endocrinol Metab (2017) 102:434–42. 10.1210/jc.2016-2965 27906594

[18] Lertrit A Chailurkit LO Ongphiphadhanakul B Aekplakorn W Sriphrapradang C. Thyroid Function is Associated With Body Mass Index and Fasting Plasma Glucose in Thai Euthyroid Population. Diabetes Metab Syndr (2019) 13:468–73. 10.1016/j.dsx.2018.11.004 30641746

[19] Chaker L Cappola AR Mooijaart SP Peeters RP. Clinical Aspects of Thyroid Function During Ageing. Lancet Diabetes Endocrinol (2018) 6:733–42. 10.1016/S2213-8587(18)30028-7 30017801

[20] Jostel A Ryder WD Shalet SM. The Use of Thyroid Function Tests in the Diagnosis of Hypopituitarism: Definition and Evaluation of the TSH Index. Clin Endocrinol (Oxf) (2009) 71:529–34. 10.1111/j.1365-2265.2009.03534.x 19226261

[21] Juiz-Valina P Cordido M Outeirino-Blanco E Pertega S Varela-Rodriguez BM Garcia-Brao MJ. Central Resistance to Thyroid Hormones in Morbidly Obese Subjects is Reversed After Bariatric Surgery-Induced Weight Loss. J Clin Med (2020) 9:359. 10.3390/jcm9020359 PMC7073690 32012985

[22] Chen Y Zhang W Wang N Wang Y Wang C Wan H. Thyroid Parameters and Kidney Disorder in Type 2 Diabetes: Results From the METAL Study. J Diabetes Res (2020) 2020:4798947. 10.1155/2020/4798947 32337292 PMC7149438

[23] Nie X Ma X Xu Y Shen Y Wang Y Bao Y. Increased Serum Adipocyte Fatty Acid-Binding Protein Levels are Associated With Decreased Sensitivity to Thyroid Hormones in the Euthyroid Population. Thyroid (2020) 30:1718–23. 10.1089/thy.2020.0011 32394790

[24] Nie X Shen Y Ma X Xu Y Wang Y Zhou J. Associations Between Thyroid Hormones and Glycated Albumin in Euthyroid and Subclinical Hypothyroid Individuals: Results of an Observational Study. Diabetes Metab Syndr Obes (2020) 13:915–23. 10.1155/2020/4798947 PMC7104252 32273743

[25] Martinez B Ortiz RM. Thyroid Hormone Regulation and Insulin Resistance: Insights From Animals Naturally Adapted to Fasting. Physiology (Bethesda) (2017) 32:141–51. 10.1152/physiol.00018.2016 28202624

[26] Yang N Yao Z Miao L Liu J Gao X Fan H. Novel Clinical Evidence of an Association Between Homocysteine and Insulin Resistance in Patients With Hypothyroidism or Subclinical Hypothyroidism. PloS One (2015) 10:e0125922. 10.1371/journal.pone.0125922 25938439 PMC4418925

[27] Xu C Zhou L Wu K Li Y Xu J Jiang D. Abnormal Glucose Metabolism and Insulin Resistance are Induced Via the IRE1alpha/XBP-1 Pathway in Subclinical Hypothyroidism. Front Endocrinol (Lausanne) (2019) 10:303. 10.3389/fendo.2019.00303 31156553 PMC6533547

[28] Maratou E Hadjidakis DJ Kollias A Tsegka K Peppa M Alevizaki M. Studies of Insulin Resistance in Patients With Clinical and Subclinical Hypothyroidism. Eur J Endocrinol (2009) 160:785–90. 10.1530/EJE-08-0797 19141606

[29] Nader NS Bahn RS Johnson MD Weaver AL Singh R Kumar S. Relationships Between Thyroid Function and Lipid Status or Insulin Resistance in a Pediatric Population. Thyroid (2010) 20:1333–9. 10.1089/thy.2010.0180 21114382

[30] Kouidhi S Berhouma R Ammar M Rouissi K Jarboui S Clerget-Froidevaux MS. Relationship of Thyroid Function With Obesity and Type 2 Diabetes in Euthyroid Tunisian Subjects. Endocr Res (2013) 38:15–23. 10.3109/07435800.2012.699987 22746188

[31] Santini F Marzullo P Rotondi M Ceccarini G Pagano L Ippolito S. Mechanisms in Endocrinology: The Crosstalk Between Thyroid Gland and Adipose Tissue: Signal Integration in Health and Disease. Eur J Endocrinol (2014) 171:R137–52. 10.1530/EJE-14-0067 25214234

[32] Lin Y Sun Z. Thyroid Hormone Potentiates Insulin Signaling and Attenuates Hyperglycemia and Insulin Resistance in a Mouse Model of Type 2 Diabetes. Br J Pharmacol (2011) 162:597–610. 10.1111/j.1476-5381.2010.01056.x 20883475 PMC3041250

[33] Furuya F Shimura H Yamashita S Endo T Kobayashi T. Liganded Thyroid Hormone Receptor-Alpha Enhances Proliferation of Pancreatic Beta-Cells. J Biol Chem (2010) 285:24477–86. 10.1074/jbc.M109.100222 PMC2915684 20529852

[34] Santiago LA Santiago DA Faustino LC Cordeiro A Lisboa PC Wondisford FE. The Delta337T Mutation on the TRbeta Causes Alterations in Growth, Adiposity, and Hepatic Glucose Homeostasis in Mice. J Endocrinol (2011) 211:39–46. 10.1530/JOE-11-0194 21746794

[35] Perry RJ Wang Y Cline GW Rabin-Court A Song JD Dufour S. Leptin Mediates a Glucose-Fatty Acid Cycle to Maintain Glucose Homeostasis in Starvation. Cell (2018) 172:234–48.e17. 10.1016/j.cell.2017.12.001 29307489 PMC5766366

[36] Mullur R Liu Y-Y Brent GA. Thyroid Hormone Regulation of Metabolism. Physiol Rev (2014) 94:355–82. 10.1152/physrev.00030.2013 PMC4044302 24692351

[37] Ortega FJ Jilkova ZM Moreno-Navarrete JM Pavelka S Rodriguez-Hermosa JI Kopeck Ygrave J. Type I Iodothyronine 5’-Deiodinase mRNA and Activity is Increased in Adipose Tissue of Obese Subjects. Int J Obes (Lond) (2012) 36:320–4. 10.1038/ijo.2011.101 21610697

[38] Jing S Xiaoying D Ying X Rui L Mingyu G Yuting C. Different Levels of Thyroid Hormones Between Impaired Fasting Glucose and Impaired Glucose Tolerance: Free T3 Affects the Prevalence of Impaired Fasting Glucose and Impaired Glucose Tolerance in Opposite Ways. Clin Endocrinol (Oxf) (2014) 80:890–8. 10.1111/cen.12384 24330392

[39] Roef GL Rietzschel ER Van Daele CM Taes YE De Buyzere ML Gillebert TC. Triiodothyronine and Free Thyroxine Levels are Differentially Associated With Metabolic Profile and Adiposity-Related Cardiovascular Risk Markers in Euthyroid Middle-Aged Subjects. Thyroid (2014) 24:223–31. 10.1089/thy.2013.0314 PMC3926145 24032604

[40] Nie X Ma X Xu Y Shen Y Wang Y Bao Y. Characteristics of Serum Thyroid Hormones in Different Metabolic Phenotypes of Obesity. Front Endocrinol (Lausanne) (2020) 11:68. 10.3389/fendo.2020.00068 32184757 PMC7058591

[41] Diez JJ Iglesias P. Relationship Between Serum Thyrotropin Concentrations and Metformin Therapy in Euthyroid Patients With Type 2 Diabetes. Clin Endocrinol (Oxf) (2013) 78:505–11. 10.1111/j.1365-2265.2012.04468.x 22686474

