# Supplementary material for: Sensitivity to Thyroid Hormones and Risk of Prediabetes: A Cross-Sectional Study
Source: Front Endocrinol (Lausanne). 2021 May 4;12:657114. doi: 10.3389/fendo.2021.657114 (PMC8129566; doi:10.3389/fendo.2021.657114)
Supplement: Supplementary file 1 [file DataSheet_1.docx]

Supplementary Material

# Supplementary Tables

Supplemental Table 1 Stratified associations for indices of thyroid hormone sensitivity and prediabetes.

|  |  | OR (95%CI) | | | | |
| --- | --- | --- | --- | --- | --- | --- |
|  | Cases/n (n%) | TSHI (+1 SD) | TT4RI (+1 SD) | TFQI (+1 SD) | PTFQI (+1 SD) | FT_3_/FT_4_ (+1SD) |
| Gender |  |  |  |  |  |  |
| Males | 816/2317 (35%) | 0.86 (0.78, 0.95) ^**^ | 0.97 (0.86, 1.09) | 0.93 (0.85, 1.01) | 0.86 (0.79, 0.94) ^**^ | 1.07 (0.98, 1.17) |
| Females | 641/2061 (31%) | 0.90 (0.82, 0.99) ^*^ | 0.81 (0.71, 0.92) ^**^ | 0.99 (0.89, 1.10) | 0.89 (0.80, 0.99)^*^ | 1.02 (0.92, 1.14) |
| *P*_for interaction_ |  | 0.43 | 0.05 | 0.37 | 0.66 | 0.51 |
| Age, y |  |  |  |  |  |  |
| ≤60 years | 1079/3655 (30%) | 0.89 (0.83, 0.96) ^**^ | 0.89 (0.81, 0.99) ^*^ | 0.94 (0.87, 1.01) | 0.87 (0.81, 0.94) ^***^ | 1.06 (0.98, 1.14) |
| >60 years | 378/723 (52%) | 0.85 (0.72, 0.99) ^*^ | 0.82 (0.67, 1.00) | 1.04 (0.90, 1.20) | 0.90 (0.78, 1.04) | 0.97 (0.84, 1.13) |
| *P* _for interaction_ |  | 0.80 | 0.78 | 0.27 | 0.43 | 0.38 |
| BMI categories |  |  |  |  |  |  |
| BMI<24.0 | 405/1656 (24%) | 0.85 (0.76, 0.95) ^**^ | 0.73 (0.59, 0.90) ^**^ | 0.91 (0.81, 1.03) | 0.83 (0.74, 0.94) ^**^ | 1.11 (0.98, 1.26) |
| 24.0≤BMI＜28 | 676/1899 (36%) | 0.89 (0.80, 0.98) ^*^ | 0.94 (0.84, 1.06) | 0.98 (0.89, 1.08) | 0.90 (0.82, 1.00) ^*^ | 1.06 (0.95, 1.17) |
| BMI≥28.0 | 376/823 (46%) | 0.94 (0.83, 1.07) | 0.91 (0.75, 1.10) | 0.95 (0.82, 1.09) | 0.89 (0.77, 1.02) | 0.97 (0.86, 1.11) |
| *P*_for interaction_ |  | 0.26 | 0.15 | 0.70 | 0.44 | 0.13 |

Logistic regression model: adjusted for age, gender, BMI, WC, hypertension and dyslipidemia.

^*^P<0.05; ^**^P<0.01; ^***^P<0.001

Supplementary Table 2 The association between quartiles of TSHI and risk or prediabetes events.

|  | Cases/n (%) | OR (95% CI) |
| --- | --- | --- |
| IFG |  |  |
| 1 | 207/1095 (19%) | 1 (Reference) |
| 2 | 202/1094 (19%) | 0.98 (0.79, 1.13) |
| 3 | 182/1095 (17%) | 0.91 (0.72, 1.14) |
| 4 | 145/1094 (13%) | 0.68 (0.53, 0.86) ^**^ |
|  |  | *P*_for trend_=0.002 |
| HbA_1c_ 5.7-6.4% |  |  |
| 1 | 326/1095 (30%) | 1 (Reference) |
| 2 | 263/1094 (24%) | 0.74 (0.61, 0.90) ^**^ |
| 3 | 270/1095 (25%) | 0.78 (0.64, 0.95) ^*^ |
| 4 | 281/1094 (26%) | 0.75 (0.61, 0.92)^**^ |
|  |  | *P* _for trend_=0.011 |
| Overall prediabetes |  |  |
| 1 | 416/1095 (38%) | 1 (Reference) |
| 2 | 357/1094 (33%) | 0.78 (0.65, 0.94) ^*^ |
| 3 | 350/1095 (32%) | 0.78 (0.65, 0.94) ^*^ |
| 4 | 334/1094 (31%) | 0.68 (0.56, 0.82) ^***^ |
|  |  | *P* _for trend_<0.001 |

Logistic regression model: adjusted for age, gender, BMI, WC, hypertension and dyslipidemia.

^*^P<0.05; ^**^P<0.01; ^***^P<0.001

Supplementary Table 3 The association between quartiles of TT4RI and risk or prediabetes events.

|  | Cases/n (%) | OR (95% CI) |
| --- | --- | --- |
| IFG |  |  |
| 1 | 213/1095 (20%) | 1 (Reference) |
| 2 | 203/1094 (19%) | 0.95 (0.76, 1.78) |
| 3 | 176/1095 (16%) | 0.84 (0.67, 1.05) |
| 4 | 144/1094 (13%) | 0.66 (0.52, 0.84) ^**^ |
|  |  | *P* _for trend_=0.001 |
| HbA_1c_ 5.7-6.4% |  |  |
| 1 | 314/1095 (29%) | 1 (Reference) |
| 2 | 283/1094 (26%) | 0.87 (0.71, 1.06) |
| 3 | 259/1095 (24%) | 0.78 (0.64, 0.95)^*^ |
| 4 | 284/1094 (26%) | 0.82 (0.67, 0.99)^*^ |
|  |  | *P* _for trend_=0.028 |
| Overall prediabetes |  |  |
| 1 | 411/1095 (38%) | 1 (Reference) |
| 2 | 372/1094 (34%) | 0.85 (0.71, 1.02) |
| 3 | 338/1095 (31%) | 0.76 (0.63, 0.91) ^**^ |
| 4 | 336/1094 (23%) | 0.71 (0.59, 0.86) ^***^ |
|  |  | *P* _for trend_<0.001 |

Logistic regression model: adjusted for age, gender, BMI, WC, hypertension and dyslipidemia.

^*^P<0.05; ^**^P<0.01; ^***^P<0.001

Supplementary Table 4 The association between quartiles of TFQI and risk or prediabetes events.

|  | Cases/n (%) | OR (95% CI) |
| --- | --- | --- |
| IFG |  |  |
| 1 | 177/1095 (16%) | 1 (Reference) |
| 2 | 177/1094 (16%) | 1.04 (0.82, 1.31) |
| 3 | 185/1095 (17%) | 1.09 (0.87, 1.38) |
| 4 | 197/1094 (18%) | 1.14 (0.91, 1.44) |
|  |  | *P* _for trend_=0.221 |
| HbA_1c_ 5.7-6.4% |  |  |
| 1 | 323/1095 (30%) | 1 (Reference) |
| 2 | 288/1094 (26%) | 0.90 (0.74, 1.10) |
| 3 | 255/1095 (23%) | 0.75 (0.61, 0.92) ^**^ |
| 4 | 274/1094 (25%) | 0.85 (0.70, 1.04) |
|  |  | *P* _for trend_=0.032 |
| Overall |  |  |
| 1 | 391/1095 (36%) | 1 (Reference) |
| 2 | 367/1094 (34%) | 0.96 (0.80, 1.16) |
| 3 | 340/1095 (31%) | 0.84 (0.70, 1.01) |
| 4 | 359/1094 (33%) | 0.93 (0.77, 1.12) |
|  |  | *P* _for trend_=0.217 |

Logistic regression model: adjusted for age, gender, BMI, WC, hypertension and dyslipidemia.

^*^P<0.05; ^**^P<0.01; ^***^P<0.001

# Supplementary Figure

**
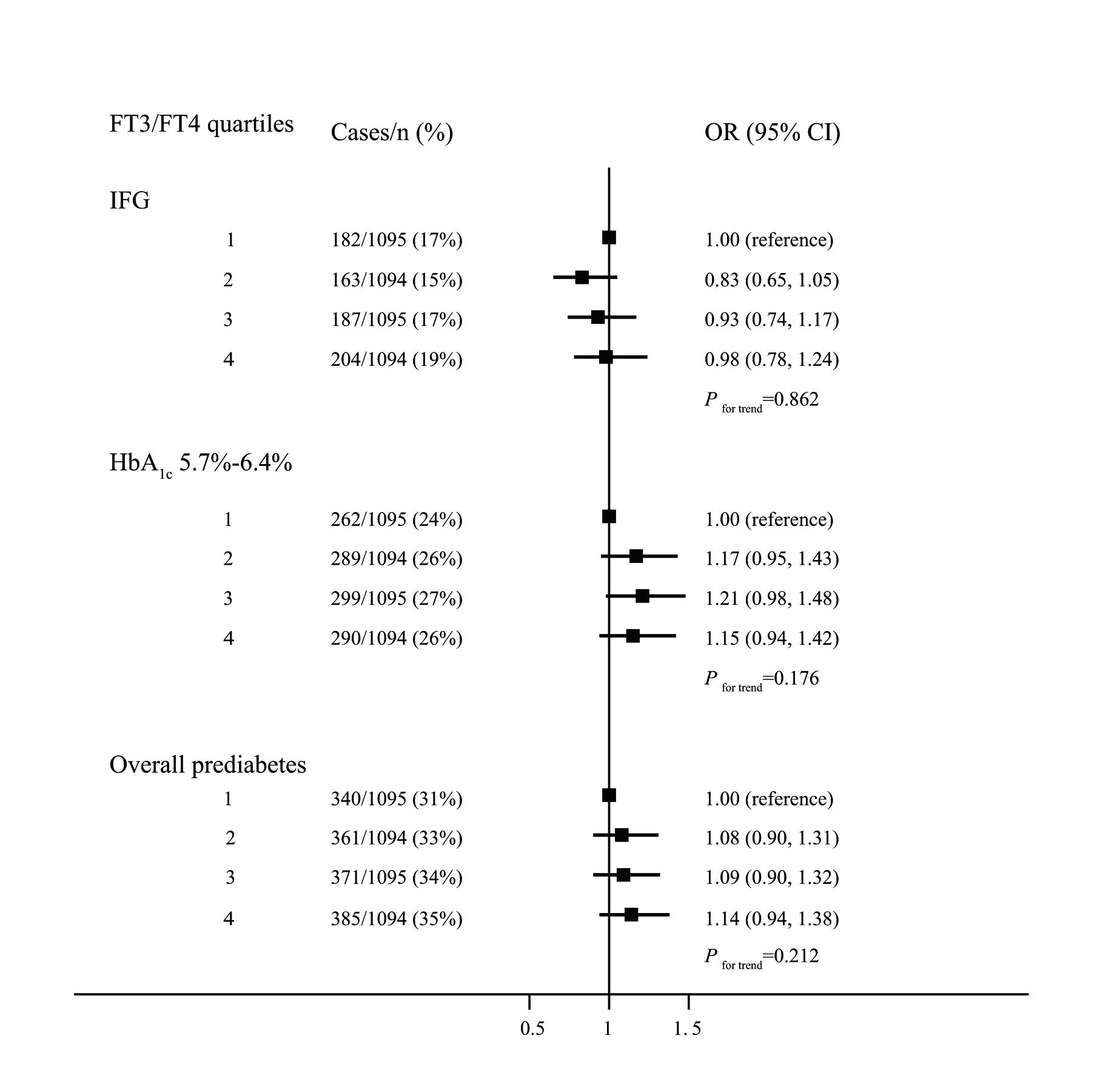
**

**Supplementary Figure 1** The association between quartiles of FT3/FT4 and risk or prediabetes. Logistic regression model; models are adjusted for age, gender, BMI, WC, hypertension and dyslipidemia.
